# Navigating the Complexities of Radiation Injuries: Therapeutic Principles and Reconstructive Strategies

**DOI:** 10.3390/jpm14111100

**Published:** 2024-11-09

**Authors:** Andreea Grosu-Bularda, Flavia-Francesca Lita, Florin-Vlad Hodea, Eliza-Maria Bordeanu-Diaconescu, Andrei Cretu, Catalina-Stefania Dumitru, Stefan Cacior, Bogdan-Mihai Marinescu, Ioan Lascar, Cristian-Sorin Hariga

**Affiliations:** 1 Department 11, Discipline Plastic and Reconstructive Surgery, University of Medicine and Pharmacy Carol Davila, 050474 Bucharest, Romania; andreea.grosu-bularda@umfcd.ro (A.G.-B.); ioan.lascar@umfcd.ro (I.L.); cristian.hariga@umfcd.ro (C.-S.H.); 2 Clinic of Plastic Surgery and Reconstructive Microsurgery, Clinical Emergency Hospital of Bucharest, 014461 Bucharest, Romania; 3 Clinical Department Plastic Surgery and Reconstructive Microsurgery, Central Military Emergency University Hospital “Dr. Carol Davila”, 010825 Bucharest, Romania

**Keywords:** radiation injuries, radiotherapy, therapeutic strategies, prevention, reconstructive surgery

## Abstract

Radiation injuries, particularly those resulting from therapeutic or accidental exposure, present complex challenges for medical management. These injuries can manifest localized skin damage or extend to deeper tissues, presenting as various clinical entities that require treatment strategies, ranging from conservative management to complex surgical interventions. Radiation treatment constitutes a fundamental component of neoplastic management, with nearly two out of three oncological instances undergoing it as an element of their therapeutic strategy. The therapeutic approach to radiation injury consists of expanding prophylactic measures while maintaining the efficacy of treatment, such as conservative treatment or local debridement followed by reconstruction. The armamentarium of reconstructive methods available for plastic surgeons, from secondary healing to free tissue transfer, can be successfully applied to radiation injuries. However, the unique pathophysiological changes induced by radiation necessitate a careful and specialized approach for their application, considering the altered tissue characteristics and healing dynamics. The therapeutic strategy is guided by both the severity and progression of the injury, with the primary aim of restoring functionality and aesthetic aspects while simultaneously minimizing the risk of complications. This paper explores the various conditions encompassed by the term “radiation injury,” reviews both non-surgical and surgical therapeutic strategies for managing these injuries, and highlights the unique challenges associated with treating irradiated tissues within specific oncological contexts.

## 1. Overview on Radiation Biology and Radiotherapy

Radiation therapy (RT) constitutes a fundamental element in the management of diverse malignancies, with approximately two-thirds of oncological patients receiving RT as an integral component of their comprehensive treatment regimens. The field of radiation oncology is dedicated to examining, preventing, and addressing neoplasms, thus underscoring the significance of ionizing radiation. The role of radiation oncologists exists within a collaborative network that brings together specialized surgical and medical oncologists, along with diagnostic radiologists and pathologists, all focused on delivering the most effective evidence-based interventions for those with cancer [1,2,3,4].

Certain benign conditions can also be effectively treated with radiation therapy, such as keloid scars, aggressive fibromatosis, including desmoid tumors, or as a preventive strategy for heterotopic ossification. These conditions involve uncontrolled cell growth that, while not malignant and unable to metastasize, can still cause harm due to excessive overgrowth. Radiation therapy can often stop the rapid growth of these cells and manage the pathological process. For all these, it is important to weigh the potential risks of radiation against the anticipated benefits, though in the majority of indicated cases, the benefits outweigh the risks [5,6,7]. Radioiodine therapy is an established treatment of benign thyroid disease, being indicated in treating hyperthyroidism in patients with Graves’ disease or toxic/nontoxic multinodular goiter and for reducing the overall volume of the thyroid gland, as in cases of Graves’ disease, non-toxic goiter, or hyperfunctioning nodules [8]. In the following sections of this paper, we will address the usefulness of radiotherapy in malignant pathology.

Significant progress has been made in recent years in utilizing ionizing radiation to treat cancer. The effects of radiotherapy depend on several factors, including the radiation dose used, the type of cancer being targeted and its radiosensitivity, the size and anatomical location of the tumor, as well as the tolerance threshold of healthy tissues to radiation (Normal Tissue Tolerance—NTT) [2,9].

Radiotherapy is often used in combination with other treatment approaches such as surgery, chemotherapy, or immunotherapy, and alongside surgery, it is one of the primary local treatment methods for tumors. When applied before surgery (neoadjuvant therapy), its purpose is to reduce the size of the tumor. When used after surgery (adjuvant therapy), it targets and eliminates any remaining microscopic tumor cells. Radiation therapy can be administered to cure cancer or as an effective palliative treatment to alleviate symptoms caused by the disease [2,3].

The Gray (Gy) is the SI unit for measuring radiation, equivalent to 1 Joule/kg of energy absorbed from ionizing radiation [9]. The radiation dose required for complete and permanent tumor regression in a specific treatment area is referred to as the Tumor Lethal Dose (TLD). The more radiosensitive a tumor is, the greater the effectiveness of radiotherapy, which can be quantified by the Therapeutic Index (TI), calculated as the ratio between NTT and TLD. It is well understood that different tumors vary in their sensitivity to radiation therapy. When the radiation dose needed to achieve tumor regression is low (low TLD) and surrounding tissues are more resistant (high NTT), the cancer is considered radiosensitive (e.g., lymphomas, seminomas, and embryonal tumors). Conversely, when TLD is high and NTT is low, the Therapeutic Index will be lower, classifying tumors as radioresistant (e.g., sarcomas, melanomas, gliomas) [2].

The challenge in RT is to correctly adapt the doses to effectively target the tumoral tissue, having also in mind to reduce the exposure to the surrounding normal tissues. Cells with high proliferation rates, such as those found in the skin, hematopoietic tissue, and gastrointestinal tract, exhibit increased susceptibility to ionizing radiation. This heightened vulnerability is attributed to their active cell division, which makes them more sensitive to the damaging effects of radiation on DNA and cellular structures [10]. However, based on their regenerative potential, after the radiation effect is overcome, these specific tissues may recover from the injury. Other tissues, such as nervous tissue, may suffer severe irreversible damage [11].

Understanding radiation toxicity is crucial in radiation oncology, with extensive literature guiding best practices [12]. RT induces cellular death by compromising genomic DNA, either directly or indirectly, through the production of reactive oxygen species. The degree of impairment and the cell’s capacity for repair are based on variables such as the cell’s differentiation state and mitotic frequency, in addition to cumulative and fractional radiation exposures. Although proteins and lipids are similarly compromised, DNA impairment is pivotal for cellular lethality, with hydroxyl radicals accounting for approximately 60–70% [13,14,15,16]. Ionizing radiation primarily targets cellular DNA, causing both double-strand and single-strand breaks, ultimately leading to cell death via apoptosis or necrosis. Apoptosis is an active process, also known as programmed cell death, where radiation-damaged cells undergo self-destruction. In necrosis, irradiated cells that progress through mitosis with unrepaired DNA lesions experience passive cell death [17,18].

Damage to the double-stranded DNA structure leads to chromosomal aberrations. Cells with such lesions form micronuclei during division, which are responsible for mitosis-linked cell death, the most common form of radiation-induced cell death, usually resulting in necrosis. Micronuclei are encapsulations of the nuclear membrane that contain fragments of chromosomes and chromatin, which become trapped during the cell cycle and are unable to reintegrate into the primary nucleus at the end of cell division. These structures are proven to be markers of genotoxic stress, which is involved in carcinogenesis, metastasis, and cellular senescence. The severity of genetic damage in cells with micronuclei often leads to their death. Cellular necrosis triggers local inflammation in patients undergoing radiotherapy, as tumor cells lose membrane integrity, swell, develop cytoplasmic vesicle dilatations, and undergo further DNA degradation [17,18,19,20,21].

Apoptosis, or programmed cell death, is marked by specific morphological changes, including nuclear chromatin condensation, membrane blebbing, and nuclear fragmentation, leading to the formation of apoptotic bodies. Biochemically, apoptosis is triggered by the activation of caspases, especially caspase-3, which cleaves various cellular proteins and DNA fragmentation factors, resulting in cytoskeletal breakdown and DNA fragmentation. The timing of apoptosis varies by cell type, occurring either immediately after radiation (fast apoptosis) or after one or more cell divisions (delayed apoptosis). Radiosensitive cells, such as thymocytes and lymphocytes, undergo rapid apoptosis, while other cells, like gastric tumor cells, display delayed apoptosis after progressing through cell cycle checkpoints. Radiation induces cell cycle arrests in the G1 and G2 phases, allowing time for DNA repair. These checkpoints, regulated by CDK-cyclin complexes, influence the likelihood of apoptosis. Shorter G2 delays after radiation are associated with higher apoptosis rates, while prolonged G2 arrest may suppress apoptosis [17,18,19,20,21].

The tumor suppressor protein p53 plays a crucial role in radiation-induced apoptosis. Cells lacking p53, such as those in p53-null mice, are resistant to apoptosis, suggesting p53 is necessary for early apoptosis. However, some cells can undergo p53-independent apoptosis, occurring later and linked to the G2/M phase. In tissues like the thymus and nervous system, both p53 and the ATM protein are involved in apoptosis, though their roles vary by tissue type [17].

The extent of radiation-induced apoptosis is also linked to radiosensitivity, with faster apoptosis correlating with higher sensitivity. Radiosensitive hematopoietic cell lines, for example, undergo quicker apoptosis than radioresistant ones, which exhibit delayed G2 arrest before apoptosis. However, this relationship may vary between cell types, as some studies suggest that delaying apoptosis does not necessarily affect long-term survival after radiation [17,18,19,20,21].

Another key structure affected by radiation is the cell membrane, which contributes directly to cell death. Ionizing radiation disrupts membrane sphingomyelins, generating ceramides. These lipid second messengers activate the c-Jun amino-terminal kinase pathway, promoting apoptosis. Ceramides also play a role in the dephosphorylation of the anti-apoptotic protein Bcl-2 through the activation of mitochondrial protein phosphatase 2A (PP2A) [17,22].

The repercussions of radiation exposure can be assessed from two main viewpoints: the interaction of radiation with cancerous cells and the negative damage it causes to healthy cells. Even though the therapeutic ratio describes the dose-response relationship between achieving tumor control and avoiding complications in normal tissues, helping to balance these two aspects, in reality, it is challenging to deliver a sufficiently high dose to eliminate tumors without causing significant harm to healthy tissues [23,24,25]. Adverse effects on healthy tissues are classified as acute or late. Acute effects occur within the first 90 days of treatment, affecting tissues with a high cell division rate, such as epithelial cells. Late effects arise after 90 days, primarily due to damage to local microvascularization or the depletion of stem cells in certain regions [2].

Radiotherapy is categorized based on its delivery method into teletherapy and brachytherapy [2,3,9,26]. Teletherapy, or External-beam radiation therapy (EBRT), is the most commonly used method and involves an external radiation source directing beams at a specific anatomical region of the patient [2,9,26]. This therapy is administered in pre-determined fractions, allowing normal cells time to recover between doses due to their superior DNA repair capacity compared to cancer cells [9,23].

Recent advances in external radiation therapy have focused on improving precision and effectiveness and minimizing side effects. A series of technological improvements are currently used, including stereotactic radiotherapy, intensity-modulated radiotherapy, three-dimensional conformal radiotherapy, and image-guided radiotherapy [27,28,29,30].

Three-dimensional Conformal Radiotherapy (3-DCRT) uses a computer-generated 3D image of the anatomical region to be treated, allowing precise adjustments to the radiation field based on the proximity of vital radiosensitive structures. This makes 3-DCRT particularly useful for treating tumors located near critical structures. Intensity-Modulated Radiotherapy (IMRT), a type of 3-DCRT, allows the modulation of both the intensity and shape of radiation beams to deliver different radiation doses to the tumor and adjacent tissues. IMRT is particularly useful for patients who have already received the maximum radiation dose through conventional methods. Gamma-knife is a form of stereotactic radiosurgery used for intracranial tumors, where multiple radiation beams focus on the tumor, delivering a high radiation dose while sparing healthy tissue. Another type of stereotactic surgery, X-knife, offers lower precision than Gamma-knife but can be used for extracranial tumors, such as those in the lungs or spine [2,9,23,26].

Recent technologies were introduced, including protons and carbon ions (hadrons) therapies, having beneficial properties for treating patients with cancer. They offer greater precision compared to conventional X-rays and have radiobiological advantages that make them effective for treating radio-resistant or inoperable tumors [31].

Hadron therapy, also known as particle therapy, involves irradiating tumors with heavy particles such as protons, alpha particles, or carbon ions. The primary advantage of this approach is its specific energy distribution in depth [32]. Unlike photons, which deliver a dose beyond the target area as they pass through the patient’s body, hadrons lose their kinetic energy and release the majority of the dose at the Bragg Peak, which is extended to match the tumor’s thickness, while depositing minimal dose beyond the tumor. This approach allows the delivery of the dose to the malignant target while better protecting the surrounding healthy tissues and also makes it possible to use equal or higher irradiation doses with a reduced toxicity profile [33]. Carbon ions have the advantage over proton therapy of reducing lateral scattering, which could further improve therapeutic results and spare the organs at risk. Furthermore, carbon ions exhibit a higher linear energy transfer, causing DNA damage clustered in a way that exceeds the cell’s repair mechanisms. This explains the greater relative biological effectiveness of carbon ions compared to protons and photons. Carbon ion therapy holds great promise for delivering high doses to targeted areas while minimizing damage to nearby organs at risk. However, access to this treatment remains limited due to its high cost [33,34].

Boron Neutron Capture Therapy (BNCT) is another emerging technique developed to optimize radiation delivery. It is based on selectively concentrating boron compounds in tumoral cells and then exposing the cancer cells to epithermal neutron beam radiation. This therapy is based on the nuclear reaction that appears through irradiation of the stable isotope of Boron-10 with low-energy thermal neutrons to generate α particles (Helium-4) and recoiling lithium-7 nuclei. The main property of BNCT is that it can deposit a high dose gradient between the cancer cells and healthy tissues. BNCT combines the fundamental targeting approach of chemotherapy with the broad anatomical localization strategy of conventional radiotherapy [35].

Brachytherapy involves placing radioactive materials inside or adjacent to the tumor. Various types of brachytherapy are used depending on the anatomical site, such as interstitial brachytherapy (radiation source placed within a parenchymal organ, e.g., prostate), intracavitary brachytherapy (source positioned within a cavity, e.g., vagina or rectum), and intraluminal brachytherapy (for organs with a lumen, e.g., esophagus). Depending on the duration the radioactive source remains in the body, brachytherapy can be classified into temporary, where the radiation source is removed after a set period, or permanent, where radioactive seeds are implanted within or near the tumor, gradually losing their radioactivity over weeks or months [2,9,23,26].

This paper aims to analyze the clinical manifestations and the available therapeutic strategies for managing radiation injuries, particularly those arising from therapeutic exposure for various malignancies. Both conservative and surgical approaches, with a focus on the pathophysiological challenges posed by radiation-damaged tissues, were analyzed. From a plastic and reconstructive surgery team point of view, we also addressed the importance of tailoring the treatment to restore function and improve the quality of life while minimizing complications, highlighting the application of reconstructive methods in the context of oncological treatment involving radiation therapy.

## 2. Clinical Manifestations and Complications Associated with Radiotherapy

Despite technological progress, radiation toxicity remains a significant issue. Acute toxicity emerges from the necrosis of rapidly proliferating cells, resulting in manifestations such as erythema and desquamation, attributable to capillary dilation and heightened vascular permeability. Late toxicity may arise from elements such as parenchymal and stromal cell depletion or ischemia-induced vascular impairment, resulting in conditions such as cutaneous fibrosis, which is characterized by hypovascular, hypoxic, and hypocellular tissue. The skin, bone, and soft tissue frequently endure considerable radiation exposure. The use of RT may cause numerous complications that can affect both malignant and healthy tissues, including both immediate and long-lasting skin conditions such as radiation dermatitis, as well as more severe issues such as osteoradionecrosis and various organ impairments. These complications can profoundly affect a patient’s quality of life, necessitating meticulous management and multidisciplinary care [11,36,37].

### 2.1. Radiodermatitis

Radiodermatitis is classified as an acute response if it manifests during therapeutic intervention and as chronic or late-onset if it arises 5–10 years after the conclusion of treatment. A joint initiative was undertaken by the Radiation Therapy Oncology Group alongside the European Organization for Research and Treatment of Cancer to establish a framework for evaluating skin condition severity damage resulting from radiation exposure. This framework sorts radiation dermatitis into four grades: grade 1 is characterized by faint erythema and dry desquamation; grade 2 is characterized by moderate to brisk erythema and patchy, moist desquamation, mostly in the skin folds; grade 3 is characterized by confluent, moist desquamation in areas other than the skin folds; and grade 4 is characterized by skin ulceration with trauma-associated or spontaneous bleeding [38].

Chronic radiodermatitis affects one-third of all individuals and may develop up to ten years after radiotherapy. Typical manifestations include telangiectasia, pigmentation alterations, cutaneous atrophy (characterized by dry, papery integument), dermal sclerosis, and keratosis. Obesity, chronic solar exposure, and tobacco use are associated with an increased risk of radiation dermatitis. Additionally, the bacterial flora of the skin, notably the presence of Staphylococcus aureus, could be associated with the onset of significant radiation dermatitis [39,40,41]. Additionally, individuals undergoing concurrent chemotherapy or targeted oncological therapies have an increased likelihood of experiencing severe radiation dermatitis [42,43,44].

### 2.2. Osteoradionecrosis

Osteoradionecrosis (ORN) is a serious complication of radiotherapy and is characterized by the gradual demise of osseous tissue, typically manifesting in skeletal structures that have been subjected to radiation. This pathology is most frequently observed in the mandible but has the potential to influence other bones exposed to radiation, such as the pelvis, ribs, or vertebrae, contingent on the treatment region [45,46].

ORN of the jaw is a late complication of RT that typically affects patients who have undergone treatment for head and neck cancers. This condition arises when previously irradiated tissues become hypovascular and hypoxic, leading to aseptic and avascular necrosis of the mandible if they fall within the radiation field. ORN can lead to important complications such as infection, tooth loss, and even pathological fractures of the mandible [47,48,49].

ORN is uncommon in patients who have received less than 6000 centi-gray (cGy) radiation but can occur years or even decades after treatment. Approximately nine percent of patients who receive > 7000 cGy of radiation to the head or neck develop this condition. Radiation exposure leaves bone and surrounding soft tissues with poor vascularity, resulting in avascular necrosis of the mandible. This can lead to areas of exposed bone in the mouth, tooth loss, and damage to supporting structures. Chronic infections often follow, potentially leading to osteomyelitis and the formation of orocutaneous fistulae [47,48].

Imaging techniques, such as radiography, panoramic imaging, computed tomography (CT), and magnetic resonance imaging (MRI), are essential for assessing the extent of ORN. However, no laboratory test can definitively diagnose ORN, except for biopsy [50,51].

Treatment of ORN requires a multidisciplinary approach involving surgery, infectious disease management, radiology, and hyperbaric treatment. These treatments must be coordinated, as isolated efforts are unlikely to succeed. Patients suffering from ORN should be staged based on severity, and if indicated, surgical intervention should be initiated in combination with perioperative hyperbaric oxygen therapy (HBOT). All necrotic bone must be surgically removed to ensure effective treatment [47,52].

Local flaps can often be used to cover the exposed bone, depending on the location and condition of the surrounding tissues. If this approach is not successful, it may be necessary to use regional or free flaps to introduce healthy, non-irradiated tissue to cover and preserve compromised bone. If these options are ineffective, segmental mandibulectomy followed by reconstruction with a vascularized bone flap may be necessary [48,53].

### 2.3. Lymphedema

Radiation treatment also presents a considerable risk of lymphedema, especially when administered to specific anatomical regions. The direct effect of radiation on lymphatic vessels is negligible, as in vitro and in vivo investigations have demonstrated that their structural and functional integrity are predominantly preserved. However, damage to lymphatic vessels occurs later as the surrounding tissue gradually becomes dense and fibrous, compressing the vessels and obstructing lymphatic flow. Additionally, RT inhibits lymphatic proliferation, prevents the growth of compensatory lymphatic vessels, and contributes to the development of lymphedema. Although lymphatic vessels are relatively resistant to radiation, lymph nodes are highly radiosensitive. Radiation causes the lymph nodes to lose lymphocytes, undergo fatty degeneration, and ultimately become fibrotic [54,55].

The risk of lymphedema is particularly pronounced in breast cancer patients undergoing RT. Johansen et al. revealed that radiotherapy in patients who underwent mastectomy increased the risk of lymphedema five-fold. This susceptibility is further exacerbated, approaching ten-fold, when radiotherapy is concomitantly administered with lymphadenectomy, with the probability escalating in accordance with the magnitude of dissection. Specific anatomical regions, such as the superior axillary nodal basins at levels I and II, exhibit an elevated risk of lymphedema owing to the heightened density of lymph nodes. However, advanced RT techniques that minimize overlapping radiation fields may reduce this risk [54,56,57,58].

RT also significantly increases the risk of lower limb lymphedema in patients with gynecological cancers such as ovarian, vulvar, and endometrial carcinomas. The method of RT delivery influences the risk, with external beam RT being associated with a higher incidence of lymphedema than vaginal brachytherapy, where radiation is delivered internally through the vagina. The estimated risk of lower limb lymphedema is 11% with vaginal brachytherapy compared to 71% following pelvic external beam RT [54,59,60,61,62].

### 2.4. Neurological Complications

Radiation therapy can lead to several neurological complications depending on the location, dose, and duration of treatment. Radiation therapy carries the risk of neural damage, which can manifest as focal cerebral necrosis, neurocognitive dysfunction, cerebrovascular disease, myelopathy, and peripheral nerve disorders, including a specific radiation-induced brachial plexus neuropathy [63,64].

Radiation-induced brain necrosis (RBN) is a significant complication that can arise following radiotherapy for both intracranial and skull base tumors. It is caused by an initial brain vascular injury followed by parenchymal damage through radiation exposure. RBN manifests with a range of neurological symptoms, including headaches, cognitive dysfunction, personality changes, and seizures, severely affecting a patient’s quality of life. The development of RBN is influenced by factors such as total radiation dose, fraction size, and the volume of brain tissue exposed to radiation. Advances in neuroimaging and histopathology have improved understanding of the condition, and while previously considered progressive and irreversible, emerging treatments, including the use of bevacizumab and nerve growth factors, offer hope for improving or reversing some cases of RBN. Nonetheless, the condition remains a challenge, and therapeutic strategies continue to evolve, balancing symptom management and functional recovery [63,65].

Other neurological complications may also occur, such as radiation myelopathy secondary to radiation exposure of the spinal cord, leading to weakness, numbness, pain, and, in severe cases, paralysis, although rare [66].

Cognitive impairment may manifest after cerebral irradiation, especially in juvenile patients or geriatric individuals, and can encompass amnesia, challenges with focus, and various cognitive shortcomings, which may progressively deteriorate over time [67].

Fatigue is a common side effect experienced by many patients undergoing RT, characterized by persistent tiredness not relieved by rest, which can last for weeks to months after treatment and can significantly impact a patient’s quality of life [68].

Radiation-induced peripheral neuropathy is a chronic and debilitating condition that is typically progressive and often irreversible, frequently emerging several years after radiotherapy. While its occurrence remains rare, it is becoming more encountered as long-term cancer survival rates improve. The underlying pathophysiological mechanisms are not yet fully understood, but a key factor is nerve compression caused by extensive radiation-induced fibrosis, along with direct nerve damage through axonal injury, demyelination, and blood vessel damage due to ischemia from capillary network alteration. The clinical presentation is highly variable, depending on the specific anatomical regions that are irradiated [69].

Radiation-induced injury to the brachial plexus (RIBPN) is an unusual and belated consequence for those who have received RT targeting the chest wall, neck, or axillary region. This ailment is predominantly observed in patients with mammary carcinoma and Hodgkin lymphoma. Advances in radiation techniques have significantly reduced the incidence of RIBPN, with a current incidence rate of approximately 1.2% in women treated for breast cancer [70,71,72,73].

RIBPN typically progresses gradually in approximately two-thirds of cases. Individuals may initially encounter paresthesia, which is followed by a dolor and ultimately motor debilitation in the impacted extremity. The onset of manifestations can fluctuate from 6 months to 20 years after RT, although it predominantly transpires between 1 and 4 years after treatment. In late-onset RIBPN, radiation-induced fibrosis compresses the nerves, leading to further complications, such as direct nerve injury from axonal damage, demyelination, and ischemic blood vessel injury. This nerve damage is chronic, progressive, and irreversible and is more common when radiation targets the axillary and/or supraclavicular nodes, as well as the breast or chest wall [70,71,72,74].

Based on the anatomy of the brachial plexus, radiation to the axillary lymph nodes primarily affects the lower trunk of the plexus (C8-T1), whereas radiation to the supraclavicular nodes tends to affect the upper trunk (C5-C6). The entire plexus is involved in approximately 25% of RIBPN cases [71,75].

As several disorders, such as carpal tunnel syndrome, thoracic outlet syndrome, and amyotrophic lateral sclerosis, can mimic the symptoms of RIBPN, it is crucial to refer patients to a neurologist for electrodiagnostic testing as soon as possible. MRI is a key diagnostic tool for RIBPN, typically showing thickening of the affected plexus nerve. Fluorodeoxyglucose PET/computed tomography (PET/CT) is recommended as an adjunct imaging modality to MRI, especially for differentiating RIBPN from neoplastic plexopathy [71,76,77,78]. According to a case report by Soydal et al., 18F-fluorodeoxyglucose PET/CT was particularly useful in distinguishing metastatic plexopathy from RIBPN, which is essential for guiding treatment decisions [79].

To address the underlying pathophysiological mechanisms of RIBPN, surgical interventions, such as plexus exploration, neurolysis with a free tissue transfer (using muscle or omentum), or pedicled omentoplasty, have shown positive outcomes [69,71].

A similar situation exists in cases of radiotherapy applied for lumbar and pelvic region radiotherapy addressing various cancer types, such as testicular cancer, and rectal or bladder malignancies getting to the development of various clinical manifestations, from acute transient lumbosacral plexopathy to severe entities such as delayed progressive lumbosacral radiculoplexopathy (RILP) and nerve trunk damage [80].

Accurate diagnosis of peripheral nerve injuries is mandatory. These cases require a dynamic, sequential evaluation that includes clinical assessment, functional testing, and imagistic studies. Despite various therapeutic strategies, both conservative and surgical, the prognosis for recovery in peripheral nerve pathology remains unpredictable. The treatment should include a comprehensive rehabilitation program under the supervision of a physiotherapist, ensuring a structured approach to recovery and optimizing functional outcomes [69,81,82,83].

### 2.5. Endocrine Effects of Radiotherapy

Endocrine complications following radiotherapy typically manifest with a delay and necessitate long-term monitoring by the radiation oncologist. Since endocrine glands are distributed throughout the body, they may be included within the radiation field of various tumors. Given that symptoms can often be subtle and non-specific, screening for these complications relies not only on targeted clinical evaluations but also on routine hormonal assessments [84].

Cranial irradiation can result in multiple late-onset endocrine effects, such as deficiencies in growth hormone (GH), thyroid-stimulating hormone (TSH), adrenocorticotropic hormone (ACTH), and gonadotropins [85,86]. The risk of these hormonal deficiencies is more closely associated with the radiation dose to the hypothalamus than the pituitary gland. The likelihood of developing growth hormone deficiency (GHD) after irradiation increases with doses of 18 Gy or higher and decreases with age [87,88,89]. Factors like pre-existing obesity, younger age at the time of irradiation (under 10 years), female sex, and prior cranial surgery elevate the risk of developing obesity following treatment [90,91]. The onset of endocrine deficiencies can vary from 3 months to more than 10 years after radiation therapy [92,93]. Cranial cancer survivors with tumors near the hypothalamic-pituitary (HP) region—or those who have undergone surgery or radiotherapy involving this area are at risk of HP dysfunction. This dysfunction can lead to deficiencies in several hormones, including growth hormone (GH), luteinizing hormone (LH), follicle-stimulating hormone (FSH), thyroid-stimulating hormone (TSH), and adrenocorticotropic hormone (ACTH). Additionally, these individuals may develop conditions such as central precocious puberty (CPP), hyperprolactinemia, and central diabetes insipidus (CDI) [94].

Growth hormone deficiency is the most common endocrine complication after cranial radiation therapy. Radiation doses exceeding 18 Gy have been associated with precocious puberty, while doses over 40 Gy may delay puberty due to gonadotropin deficiency [59,95,96].

Central hypothyroidism resulting from cranial radiation therapy is primarily due to deficiencies in thyrotropin-releasing hormone and thyroid-stimulating hormone in children who have received radiation doses exceeding 40 Gy. Primary hypothyroidism, hyperthyroidism, hyperparathyroidism, and secondary thyroid cancers are also associated with neck or mantle irradiation of >20 Gy [97,98,99,100,101,102].

Radiation doses exceeding 40 Gy may affect the adrenocorticotropic hormone (ACTH) axis to varying degrees, leading to central adrenal insufficiency [103].

High-dose cranial radiation therapy exceeding 40 Gy may predispose a child to developing hyperprolactinemia, which can interfere with the pulsatile secretion of gonadotropin-releasing hormone [104].

Cranial radiation therapy can lead to weight management issues, often intensified by concurrent deficiencies in growth hormone and thyroid function. Females, children under 4 years old at the time of treatment, and those who have received hypothalamic radiation doses exceeding 18 Gy are particularly at risk [105,106].

Radiation doses exceeding 10 Gy to the thyroid region can lead to hypothyroidism and, in rare cases, hyperthyroidism. Exposure of the thyroid gland to radiation doses above 20 Gy can increase the risk of developing thyroid nodules, making annual thyroid palpation during physical examinations important. Radiation to the neck may also lead to thyroid cancer. Recent evidence suggests that the risk of thyroid cancer rises with radiation doses up to 30 Gy but decreases with higher doses [102,107].

The impact on fertility is another highly important aspect for patients requiring radiotherapy. Hypogonadism risk is influenced by age at tumor diagnosis, pubertal status, treatment type, and dosage [108,109]. It can be central, resulting from gonadotropin deficiency after cranial irradiation doses exceeding 30 Gy, or primary, due to direct gonadal toxicity from gonadal irradiation or chemotherapy, which leads to elevated gonadotropin levels [110,111]. Hypogonadism negatively affects pubertal timing, bone mineral density, sexual function, fertility, quality of life, and both biological and psychological health [112]. In males, the testes are highly sensitive to radiation. Germ cells are damaged at lower radiation doses than Leydig cells. The effects are dose-dependent: at 1–3 Gy we have azoospermia (absence of sperm), which may be reversible; at 3–6 Gy the reversibility of azoospermia is less likely, and at over 6 Gy permanent azoospermia is likely. In doses over 20 Gy Leydig cell damage may occur, affecting testosterone production [113]. In females, total body irradiation, as well as abdominal, pelvic, and lumbosacral spine radiation—especially in postpubertal individuals treated with doses over 10 Gy—can compromise ovarian function. Oocyte depletion or damage reduces sex hormone production and fertility [114,115]. The incidence of nonsurgical premature menopause among cancer survivors is 8%, compared to less than 1% in siblings. Treatment for gonadal failure involves hormone replacement therapies [116]. Infertility due to gonadotropin deficiency can be treated with gonadotropin replacement therapy [117]. However, infertility resulting from direct gonadal damage or uterine injury is more difficult to address [110,118]. For fertility preservation in women, established methods include oocyte and embryo cryopreservation. Techniques like ovarian transposition and ovarian shielding can reduce radiation exposure [110]. In men, sperm cryopreservation before cancer treatment is the only established method for fertility preservation in pubertal and adult males [119,120,121].

### 2.6. Digestive Complications of Radiotherapy

Radiation-related gastrointestinal (GI) side effects are categorized as acute or late. Acute toxicity, such as diarrhea and nausea, occurs within three months of treatment and affects tissues with rapid cell turnover. Late toxicity arises more than three months post-treatment, affecting slower-renewing tissues, and can lead to complications like ulcerations, strictures, and obstructions [122]. Gastrointestinal symptoms are more frequent in overweight, smokers, and physically inactive men [123].

Acute radiation esophagitis typically develops within 2–3 weeks after initiating radiation therapy to the esophagus, resulting from damage to the basal epithelial layer. Patients may experience dysphagia (difficulty swallowing), odynophagia (painful swallowing), and substernal discomfort. These symptoms usually resolve within three weeks after completing treatment [124]. Management involves topical anesthetics, analgesics, antacids, promotility agents, dietary modifications, and treatment for candida infections. Oesophageal strictures, which are late effects appearing around six months post-therapy due to fibrosis and scarring of the esophageal muscles, are commonly treated with endoscopic dilation; tube feedings or gastrostomies are rarely necessary [125]. The incidence of dysphagia caused by stenosis ranges from 0.8% to 30% but is generally less than 2% for radiation doses under 50 Gy [126,127]. Treatment options for symptomatic or unresolved cases include surgical resection, placement of self-expandable metal stents, and endoscopic or fluoroscopic balloon dilation. However, there is a recurrence rate of about 40% [128,129].

Fistulas, which are abnormal connections that can be esophago-mediastinal, esophago-tracheal, or aorto-esophageal, occur in 5–10% of patients and are associated with high mortality due to infections, malnutrition, and acute complications like massive bleeding. Surgical intervention is the primary management strategy, but the prognosis remains poor [130,131].

Esophageal perforations have an incidence of approximately 5.6%, typically occurring around 14 weeks after treatment at the site of the primary tumor. They carry a high mortality rate of 21–24% for both spontaneous and iatrogenic cases [130,132]. Symptoms are often nonspecific and may include vomiting (84%), chest pain (79%), epigastric pain (47%), dysphagia (21%), and hematemesis (11%). Initial treatment includes fasting, proton pump inhibitors, and broad-spectrum antibiotics, but these measures fail in about 20% of cases. Additional surgical or endoscopic procedures may be necessary, such as endoprosthesis placement, tissue gluing, or clipping. Delays in diagnosis exceeding 24 h are associated with increased mortality [133].

Acute Radiation Enteritis typically develops within two weeks after radiation exposure to the digestive tract. It causes epithelial denudation, microabscess formation, and mucosal ulceration, leading to fluid and nutrient loss and potential bacterial translocation [134]. Diarrhea often begins during the third week of treatment, affecting 20% to 70% of patients, and usually resolves within 2 to 6 weeks after completing radiation therapy [135].

Chronic Radiation Enteritis has been described to occur in up to 20% of patients receiving pelvic radiotherapy [136]. Late radiation effects generally manifest 8 to 12 months after treatment but can appear years later in some cases. Symptoms include malabsorption, nausea, diarrhea, bleeding, abdominal pain, bloating, and fever due to abscess formation. Severe cases may lead to intermittent partial or complete small bowel obstruction. While the large intestine is less sensitive to radiation, patients can develop pancolitis, which can mimic inflammatory bowel disease [137,138].

Chronic radiation proctitis results from progressive epithelial atrophy and fibrosis associated with chronic mucosal ischemia. It typically has a delayed onset of 9 to 14 months following radiation exposure and is not linked to acute proctitis [139]. The overall rate of late complications from radiation therapy ranges between 5% and 20%, including bleeding, anemia, strictures, fistulas, and anorectal dysfunction [140].

Chronic bleeding is a common late gastrointestinal side effect of pelvic radiation therapy, occurring in 30–50% of patients [141]. However, bleeding severe enough to impair quality of life affects less than 6% of patients [142]. In most cases, the bleeding resolves spontaneously within weeks to months, and only 1–5% of patients develop severe anemia requiring transfusions [143]. Metronidazole and sucralfate enemas administered over four weeks are proven treatments that have demonstrated benefits in randomized clinical trials for chronic rectal bleeding [144]. Hyperbaric oxygen therapy is another alternative management option [145,146]. Due to the high risk of complications following radiation therapy, surgery is generally avoided. Endoscopic treatments—such as argon plasma coagulation, formalin solution application, radiofrequency ablation, cryotherapy, and rectal ligation—are commonly used. Stem cell therapy has also shown efficacy in treating refractory rectal bleeding [147].

Other complications include chronic ulcerations, anal strictures, stenosis, or fistuae. A total of 2.9% of patients had to undergo abdominoperineal resection or colostomy due to late, debilitating complications, according to the final analysis of the UNICANCER ACCORD 03 randomized trial [148].

Patients with mild symptoms generally do not require specific treatment. While stool softeners, sucralfate, sulfasalazine, and corticosteroid enemas are safe to use in these cases, they have not demonstrated significant benefits. For those who do not respond to initial therapies, surgical or endoscopic interventions are available. Hyperbaric oxygen therapy has shown effectiveness when other treatments fail, but it is expensive and not widely accessible [146].

Radiation-induced liver disease (RILD) is the most significant complication of liver irradiation, typically manifesting between two weeks and seven months after completing radiation therapy. Recent reviews report that RILD occurs in 6% to 66% of patients who receive liver radiation doses up to 30–35 Gy [149,150]. Classic RILD develops in previously healthy livers and is characterized by abdominal pain, non-icteric hepatomegaly (enlarged liver without jaundice), and ascites. The underlying pathophysiology involves veno-occlusive disease due to radiation-induced fibrosis and venous obliteration [151]. Biliary tract strictures after radiation therapy are rare and usually occur in the portion of the biliary tract affected by the initial tumor [152]. Dose recommendations for whole-liver radiation therapy are less than 30 Gy for primary liver tumors and less than 28 Gy for liver metastases [151,153]. Factors such as preexisting liver dysfunction, hepatitis B virus (HBV) infection, previous arterial chemoembolization, and portal vein thrombosis can influence the appropriate treatment dosage [154,155,156].

Radiation injuries to the pancreas can lead to a wide range of side effects due to its proximity to other organs. These side effects may include gastro-duodenal ulcers and gastrointestinal bleeding. The acinar cells of the pancreas are more sensitive to radiation than the endocrine islet cells [157]. Symptoms can include abdominal pain, malabsorption, pale and greasy stools, and weight loss. Recovery of pancreatic function after radiation injury has been reported [158]. Additionally, radiation-induced diabetes mellitus has been observed in several retrospective studies [159].

Dosimetric studies have shown that advanced radiation therapy techniques can reduce the dose to nearby organs at risk, such as the small bowel and rectum, resulting in fewer side effects [160]. Specifically, Intensity-Modulated Radiation Therapy (IMRT) has been found to deliver lower radiation doses to these organs compared to conventional three-dimensional conformal plans. Adaptive radiotherapy for both photons and protons may further decrease exposure to organs like the bladder, bowel, and rectum [161,162]. Image-guided radiation therapy is another technique that helps reduce doses to surrounding organs and minimizes gastrointestinal toxicity [163,164].

### 2.7. Renal and Genito-Urinary Tract Complications

Radiotherapy, with or without chemotherapy, used for treating abdominal and pelvic malignancies such as gastrointestinal cancers, gynecologic cancers, lymphomas, and sarcomas of the upper abdomen, as well as during total body irradiation (TBI), can lead to radiation-induced kidney injury, particularly radiation nephropathy (RN). The incidence of clinical radiation nephropathy has risen with the use of total body irradiation (TBI) in preparation for bone marrow transplantation (BMT) and as a result of radionuclide therapies [165].

The clinical progression of radiation nephropathy (RN) can be divided into acute and chronic phases. Aside from extremely high radiation doses above 50 Gy, which are not relevant to human experience, no symptoms or clinical signs typically appear during the first six months post-irradiation, a period known as the latent phase of RN. Clinical manifestations typically begin during the acute phase, occurring between 6 and 18 months post-irradiation, with initial signs often appearing between 6 and 13 months (average 8.5 months). Chronic kidney damage becomes clinically apparent more than 18 months after radiation therapy [166].

BMT-associated nephropathy typically develops gradually over several years and presents with symptoms such as proteinuria, hypertension, and impaired urine concentration [165].

Radiation therapy most frequently causes damage to the bladder and ureters. The most common complications include hemorrhagic cystitis, urethral and ureteral strictures, urinary fistulae, and the development of secondary primary malignancies [167]. Radiation cystitis is an inflammatory response in the urinary bladder induced by RT in the pelvic area, culminating in manifestations such as urinary frequency, urgency, dysuria, and hematuria. Persistent radiation cystitis may precipitate enduring impairment of the bladder epithelium, culminating in chronic symptoms and an augmented risk of bladder carcinoma [168,169].

Less common effects include erectile dysfunction, infertility, lower urinary tract dysfunction, bladder fibrosis, and necrosis [167]. Sexual dysfunction is another serious complication that manifests in male patients with erectile dysfunction due to damage to the nerves and blood vessels, whereas in women, it may result in vaginal dryness, stenosis, or dyspareunia. Higher radiation doses may be associated with a risk of developing vaginal stenosis [170].

### 2.8. Pulmonary Complications

Radiation-induced lung injury (RILI) represents an inflammatory reaction in the lungs that typically occurs after radiation to the chest area, usually for a thoracic tumor. The reasons for the wide range in RILI severity and the mechanisms driving its development are not yet fully understood [171]. It has two different evolutive stages, each of them with specific molecular characteristics and cellular changes: an early stage (radiation pneumonia, RP) and a late stage (radiation lung fibrosis, RLF). Approximately 10% to 20% of patients exhibit signs of RILI with varying degrees of severity [171]. Symptoms such as thoracalgia, fatigue, coughing, and increased heart rate might occur in any of the two stages, while fever is more common in RP than RLF. Imaging in RP typically reveals diffuse ground-glass opacity or consolidation in the area of radiation treatment, often accompanied by bronchial pulling and fibrotic changes. Cross-sectional imaging in RLF usually displays areas of consolidation in the lungs, ventilated bronchial signs, strip shadows, or honeycomb-like alterations in the area where the lung tissue had been irradiated. The most widely used classification system for RILI is the Common Terminology Criteria for Adverse Events, where it is graded based on symptoms, degree of fibrosis, and the need for intervention. It is often associated with bacterial, viral, or fungal infections, such as Pneumocystis carinii, and is treated concurrently with antibiotics [171,172,173].

### 2.9. Cardiovascular Complications

Cardiovascular complications, called Radiation-Induced Heart Disease (RIHD), may develop following RT to the chest. Complications that may arise include pericarditis, coronary artery pathology, valvular impairment, arrhythmias, and cardiomyopathy [174]. The risk of RIHD increases with higher radiation doses and proximity of the heart to the radiation field. There is ongoing debate regarding the safest radiation dose, which cardiac substructures are most sensitive to RT-induced injury, and the most appropriate strategies to minimize RT-related CVD [174,175,176].

According to the European Society of Cardiology Guidelines on cardio-oncology, it is recommended to categorize the risk of radiotherapy-induced cardiovascular toxicity based on mean heart dose (MHD) rather than the prescribed dose, as the latter may not accurately represent the actual radiation exposure to the heart. However, MHD is not a flawless measure, as in some cases, a small area of the heart may receive a very high dose, posing a significant risk even when the overall MHD is low. Primary prevention of RIHD relies on technological advancements that improve the precision of the radiation targeting. Current strategies aim to reduce the mean heart dose (MHD) by tailoring the distribution of the established dose or by using various strategies of respiratory management. However, it is not always feasible to perfectly avoid the heart, as the tumor is often in its proximity, such as central lung tumors, mediastinal lymphomas, or when irradiating the internal mammary chain in breast cancer treatment. When it comes to secondary preventative measures to minimize the risk of cardiovascular events, currently there are none, but, due to the established role of conventional cardiovascular risk factors in RT-related events, adjusting the modifiable risk factors is recommended for all patients before and after RT [177,178].

Coronary heart disease is nowadays increasingly treated interventionally, but this approach has its challenges when it comes to radiation-induced coronary heart disease (RICHD), as does the classic revascularization through open surgery [179]. Surgical revascularization in patients with RICHD is often challenging due to factors like poor tissue healing, radiation-related lung injury, limited bypass targets, and the need for complex procedures. Furthermore, the internal mammary artery is prone to radiation damage, making careful angiographic evaluation essential before grafting [180,181]. Coronary artery bypass grafting in this population carries higher risks of graft failure, perioperative complications, and increased all-cause mortality. When revascularization is needed, percutaneous coronary intervention (PCI) with drug-eluting stent (DES) placement is generally the preferred and safest option. Extended dual antiplatelet therapy should also be considered for patients undergoing bare metal stenting or balloon angioplasty, when appropriate. In cases where both critical native vessel disease and valvular abnormalities are present, a combined approach using PCI with DES placement and transcatheter valve repair may be worth considering as an alternative to surgery [181].

### 2.10. Effects on Bone Marrow

Another significant adverse effect of radiation therapy is the impairment of bone marrow function. When radiation targets bone tissue, it can alter the skeletal structure and lead to a depletion of cells in the bone marrow. This increases the risk of fractures in weakened bones and reduces the number of peripheral blood cells [182].

Ionizing radiation reduces the number of osteoblasts (the cells responsible for bone formation) while increasing the number of osteoclasts (the cells that break down bone), resulting in changes to the trabecular bone structure. Certain radioactive isotopes, such as strontium-89 and radium-226, have a similar structure to calcium ions, which allows them to be incorporated into the bone. This increases toxicity at this level [183,184]. Additionally, fat cells can invade the bone marrow cavity, replacing hematopoietic (blood-forming) cells with adipocytes (fat cells) [185].

The degree of direct damage to the bone marrow depends on various factors, including the percentage of marrow that is irradiated, the dose and fractionation of radiation therapy, and whether radiation is used in combination with chemotherapy. Hematopoietic cells are generally more vulnerable than mesenchymal cells [182,186].

At the level of hematopoietic stem cells, ionizing radiation can induce cell death (apoptosis), aging (senescence), or stimulate their differentiation into various types of blood cells. When stem cells are pushed to differentiate too aggressively, their ability to renew themselves diminishes, leading to bone marrow insufficiency. The lymphohematopoietic lineage is typically the first to be affected, with circulating lymphocyte levels potentially dropping significantly at doses as low as 0.3 Gy. This reduction in lymphocytes can impair immune function, posing a life-threatening risk associated with radiation therapy [186].

Hematologic toxicity is more common in patients receiving chemotherapy who also undergo radiation therapy in the pelvic area. These patients may experience symptoms like fatigue, bleeding, and an increased risk of infections, which can lead to longer hospital stays and delays in their treatment schedules. Hematoietic syndrome is characterized by low levels of lymphocytes, platelets, and granulocytes. This syndrome can occur with radiation exposure greater than 1 Gy, and at doses between 4.5 and 6 Gy, severe bleeding or infections may result in death if timely medical intervention is not provided [186].

In summary, ionizing radiation negatively impacts both the quality of bone by altering its trabecular structure and the hematopoietic function of the bone marrow. This increases the risk of fractures in weakened bones and can lead to life-threatening conditions like pancytopenia [182,183,184,185,186].

### 2.11. Radiation-Induced Malignancies

One of the most consequential effects of radiotherapy on normal tissues is the provocation of mutations, which may result in radiation-induced neoplasms [187,188]. Radiation can lead to secondary malignancies, with the absolute risk for cancer survivors following radiotherapy ranging from 0.2% to 1% per year. The occurrence of radiation-induced secondary malignancies follows a bimodal pattern. The first peak typically appears within three years of radiation exposure, predominately after hematological cancers such as acute leukemias. The second peak occurs more than ten years after treatment, mainly involving solid tumors [11,189]. Table 1 depicts different associations between primary neoplasms and secondary radiation-induced malignancies.

Subsequent malignant neoplasms (SMNs) are complications that emerge after exposure to genotoxic interventions such as radiotherapy and specific chemotherapeutic substances. Survivors of pediatric cancers are particularly at risk of developing second and even third cancers, including multiple and distinct SMNs. The underlying reasons for this increase in susceptibility remain poorly understood. Among these survivors, brain tumors are notable SMNs, with meningiomas being the most common central nervous system tumor, followed by high-grade gliomas [187,188,190,191].

Hodgkin disease (HD), a malignancy involving the lymph nodes, is typically treated with a combination of chemotherapy and radiotherapy. HD often affects the cervical and mediastinal lymph nodes, and traditional radiotherapy for HD targets these regions, inadvertently exposing the mammary tissues and lungs to radiation. The classic mantle field technique developed decades ago to treat the nodal regions involved in HD involves fractionated radiation directed at the cervical, supraclavicular, infraclavicular, and mediastinal lymph nodes. Consequently, survivors have heightened susceptibility to radiation-induced neoplasms, including mammary, pulmonary, and thyroid malignancies [190,192,193].

Radiation-induced breast cancer can also occur following radiotherapy for primary breast cancer, particularly when tangential irradiation causes radiation scattering in the contralateral breast. Women diagnosed with mammary cancer have an additional approximately 50% augmented probability of manifesting a secondary neoplasm, primarily attributable to malignancy in the contralateral breast. Furthermore, breast irradiation has been correlated with an increased risk of pulmonary cancer, although contemporary radiotherapeutic methods may help mitigate this risk [187,194].

The development of hematologic malignancies after low-dose radiation exposure has been attributed to the unique sensitivity of bone marrow cells from which leukemia originates. Higher radiation doses are thought to eliminate these cells, thereby preventing mutagenesis from manifesting as a future disease [187].

## 3. Therapeutic Management

The management of complications resulting from RT requires a multifaceted approach that combines appropriate planning, prophylactic strategies, early identification, and targeted interventions [195]. Alternative therapeutic radiation treatment modalities, such as intensity-modulated radiation therapy (IMRT) or proton therapy, can be used to accurately target tumors while minimizing radiation exposure to adjacent healthy tissues. [30,196,197] Supportive care, such as using pharmacological agents to alleviate symptoms like pain, inflammation, or nausea, helps to increase patient comfort during and post-treatment. Rehabilitation services, including physical rehabilitation, speech rehabilitation, or work therapy, play a crucial role in mitigating functional difficulties stemming from radiation-related injuries, striving to enhance the patient’s quality of life and overall capabilities [198,199].

In cases where RT causes significant tissue damage or deformities, the role of the reconstructive surgeon is essential. Reconstructive surgeons aim to repair or reconstruct damaged tissues, such as the skin, bones, or soft tissues, that have been compromised by radiation exposure. This may involve complex surgical procedures such as flap reconstruction, including bone or composite tissue transfer, which helps restore form and function to affected areas, enabling patients to perform daily activities with greater ease and independence while also contributing significantly to their psychological well-being [200,201,202].

This collaborative approach, integrating surgical expertise with radiation oncology and supportive care, helps ensure the comprehensive management of radiation-induced complications and optimizes patient outcomes [203].

### 3.1. Preventive Strategies

A comprehensive preventive protocol for minimizing radiotherapy toxicity includes a series of strategies designed to protect healthy tissues and optimize patient outcomes.

**Figure 1:**
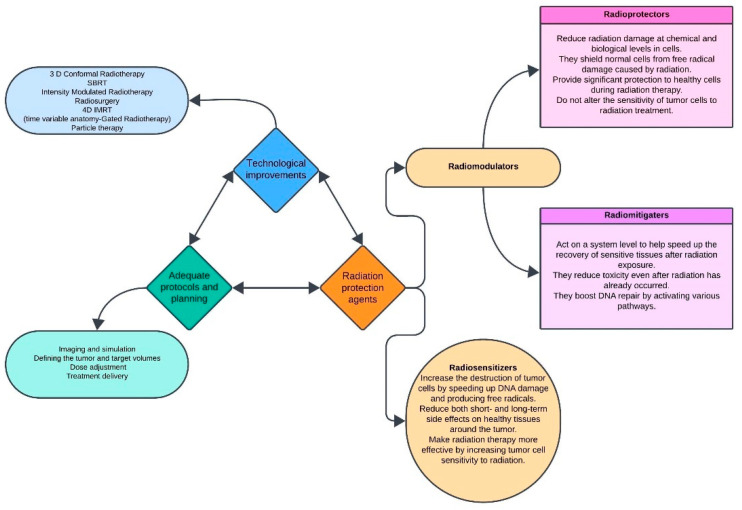
Preventive strategy in radiotherapy [204,205,206,207]. SBRT—stereotactic body radiation therapy; IMRT—intensity-modulated radiation therapy.

Figure 1 presents the components of the preventive strategy in radiotherapy, whose combination leads to risk reduction and optimization of patient recovery.

A thorough pretreatment evaluation should be conducted to identify patient-specific risk factors for chemotherapy and radiotherapy toxicity, such as pre-existing comorbidities, nutritional status, and genetic predispositions. Interventions may include optimizing nutrition, correcting anemia, managing pre-existing conditions, such as diabetes or cardiovascular disease, and encouraging smoking cessation, as these factors can influence the severity of radiation-induced side effects [208,209].

Educating patients about the potential side effects of RT, self-care practices, and early signs of complications ensure prompt reporting and management of toxicity. This engagement can help mitigate long-term side effects and improve adherence to supportive care [210].

Advancements in radiotherapy techniques have a significant role in minimizing harm to surrounding normal tissues. Over the past century, radiotherapy has developed significantly, benefiting from key innovations like 3D-Conformal Radiotherapy, Intensity-Modulated Radiotherapy (IMRT) and particle therapies that enhanced the precision of tumor targeting, reducing side effects, and improving patient outcomes [206].

Volumetric Modulated Arc Therapy (VMAT) is an advanced form of intensity-modulated radiation therapy that, unlike traditional IMRT that delivers radiation from multiple fixed angles, is continuously rotating and administers radiation from a full 360° beam angle. During this rotation, both the radiation dose rate and the shape of the radiation beam can be adjusted, allowing for more efficient and accurate targeting of complex tumor shapes. This technique not only improves dose delivery but also reduces treatment duration, increases patient comfort, and reduces the radiation dose to the rest of the body. VMAT is used for various cancers, including head and neck, brain, thoracic, pelvic, and prostate cancers, due to its ability to optimize dose delivery while minimizing side effects [211,212].

In Japan, a drug named borofalan (^10^B), along with a therapeutic system and a dose calculation program for boron neutron capture therapy (BNCT), were approved in March 2020 to treat unresectable head and neck carcinoma. BNCT involves administering borofalan (^10^B) intravenously, followed by neutron irradiation, triggering a nuclear reaction that selectively destroys tumoral cells. Clinical trials showed an overall response rate of 71.4% in patients with advanced or recurrent cancers that could not be managed by standard therapies. The treatment demonstrated high tumor selectivity and minimal invasiveness, maintaining patients’ quality of life. However, it carries risks like dysphagia, brain abscess, hemorrhage, cataract, and skin disorders, necessitating careful monitoring and further studies [213].

Radiation treatment planning should incorporate imaging techniques, such as CT, MRI, or PET scans, to accurately define tumor volume and critical organs at risk. The use of techniques such as image-guided radiation therapy (IGRT) and respiratory gating helps in real-time adjustment of radiation delivery to account for patient movement, breathing, and other physiological changes. This reduces radiation exposure to organs at risk, such as the heart, lungs, and kidneys, especially during thoracic or abdominal irradiation [214,215].

Fractionation schedules reflect the way in which radiation doses are divided and delivered over time. Currently, these schedules aim to optimize tumor control while minimizing damage to surrounding normal tissues. Different fractionation schedules have been proposed to address various clinical situations and cancer types [206,216].

Accelerated fractionation is obtained by reducing the overall course of a radiotherapy regimen without significantly diminishing the size of dose per fraction or total dose. In this approach, two or more fractions are given on some or all of the treatment days. Accelerated hyper-fractionated regimens are designed to shorten the total treatment duration while also lowering the dose per fraction. This approach aims to achieve therapeutic benefits by limiting the regrowth of tumor clonogens, an important aspect in rapidly growing tumors, while minimizing damage to late-responding normal tissues [217].

CHART (Continuous Hyperfractionated Accelerated Radiotherapy) represents an aggressive fractionation schedule that involves giving small doses of radiation three times a day, daily, over a continuous period (usually approximative 12 days). The CHART protocol significantly reduces the overall treatment duration (from about 6–7 weeks in conventional therapy to just 12 days) without increasing the overall dose per fraction. This approach helps to reduce the chances of tumor cell repopulation and is particularly used in non-small cell lung cancer [217,218].

Adaptive radiation therapy (ART) adjusts the therapeutic plan based on real-time changes in the patient’s anatomy, tumor size, shape, or response over the course of therapy. Imaging and assessment are conducted regularly during the treatment, therefore allowing the refinement of the treatment to better target the tumor while minimizing exposure to healthy tissues. For example, if a tumor reduces in size, the remaining doses are adapted to focus on the smaller area, enhancing precision and reducing side effects [219,220].

Participation in clinical trials allows for the exploration of new radiotherapy techniques, dose optimization, and development of novel protective agents. Continuous research is essential to refine the existing protocols and discover new strategies to reduce radiation-induced toxicity [203]. As an example, flash radiotherapy (FLASH-RT), an innovative approach to radiotherapy that delivers radiation doses in a short timeframe of milliseconds, has shown potential in preclinical studies to spare normal tissues while maintaining tumor control, offering a promising alternative to conventional radiotherapy with reduced side effects [221]. Dose reduction for certain types of sarcomas, such as liposarcoma, has been documented during preoperative radiotherapy, maintaining oncological outcomes while reducing morbidity, particularly wound complications [222]. By employing these strategies, a preventive protocol can effectively minimize the adverse effects of radiotherapy, improve patient quality of life, and optimize therapeutic outcomes.

Radioprotectors are administered before exposure to radiation to protect normal cells from radiation side effects. These agents are delivered either before or at the time of irradiation with the aim of preventing acute and chronic damage to normal tissues [223].

Radioprotectors have various mechanisms of action, the most common of them being free radical scavenging, increasing antioxidant levels, enhancing DNA damage repair, blocking apoptosis and growth factors, cytokines, and redox gene modulation [205].

Early uses of radioprotectors focused on synthetic thiol-containing molecules, such as WR-2721 (amifostine), which was particularly used with the purpose of protecting the salivary glands during head and neck radiotherapy and of reducing xerostomia through its free radical scavenging properties. Although it is one of the only two radioprotectors approved by the Food and Drug Administration (FDA) of the United States of America, it has significant limitations due to its potential toxicity and delivery requirements. Thus, it is falling out of use, especially when several studies seem to have different results when assessing its efficacy levels [205,224,225].

Besides thiol-containing molecules, other protective agents are represented in Table 2, although most of them are not commonly used and some of them are still studied in pre-clinical or clinical trials. The only two agents officially approved by the FDA as radioprotectors are amifostine and palifermin. The latter is a recombinant keratinocyte growth factor, although it can also be classified as a radiomitigator due to its efficiency in both preventing and treating oral mucositis [205,226,227].

Radiomitigators are administered after exposure to radiation to reduce the adverse effects. These agents are administered at the time of or after irradiation has been completed, but before the onset of normal tissue toxicity. Their purpose is to reduce the impact of radiation on normal tissues before symptoms emerge, a phenomenon often referred to as radiation emergence syndrome [205,223].

There are four agents approved by the FDA as radiomitigators: filgrastim (human recombinant G-CSF), pegfilgrastim (long-acting PEGylated form of the recombinant human G-CSF), sargramostim (recombinant GM-CSF), and, most recently, romiplostim (fusion protein analog of thrombopoietin) [205]. The mechanism of action of the G-CSF derived growth factors (filgrastim and pegfilgrastim) is the promotion of both proliferation and differentiation of myeloid progenitor cells, counteracting the neutropenia occurring after radiotherapy. Similarly, sargramostim has the same effects on macrophage and granulocyte progenitor cells, decreasing the rate of infection. Notably, sargramostim was observed to have antifibrotic properties in RILI [205,223].

Radiosensitizers are employed during radiotherapy to increase tumor cells susceptibility to radiation. These chemical or pharmaceutical agents are administered during treatment to improve the killing effect on tumor cells by speeding DNA damage and indirectly producing free radicals. Radiosensitizers can also be applied in cases of accidental radiation exposure and are useful for treating acute or chronic late effects following radiation therapy [205].

Incorporating proactive supportive care measures, such as antiemetics, antidiarrheal agents, and skin care regimens, helps manage acute side effects and reduce the risk of chronic complications. Regular follow-up and early intervention strategies for side effects, such as mucositis, dermatitis, or lymphedema, are crucial for preventing severe toxicity [228,229].

### 3.2. Therapeutic Strategy—Conservative Approach

Early intervention with conservative methods can be beneficial, especially in the initial stages of radiation injury, such as dermatitis, which is characterized by atrophy and fibrosis without tissue breakdown. The conservative approach follows two directions: symptomatic relief and pain management, with local care and systemic treatment, and prevention of complications and promotion of wound healing through nonpharmacological and pharmacological measures [230,231]. Prevention of infection is essential, particularly in instances where the integrity of the skin barrier is undermined. This necessitates the application of localized antiseptics and antibiotics to avert and manage infections within tissues that have undergone radiation-induced damage. In addition, topical agents such as ascorbic acid, pantothenic acid, and triethanolamine cream, in addition to regular cleaning and dressing of wounds, are necessary to promote healing, prevent further tissue damage, support tissue regeneration, and minimize fibrosis [36,230].

Non-pharmacological interventions such as HBOT may augment wound healing in tissues compromised by radiation through the elevation of oxygen supply, thereby facilitating angiogenesis and stimulating fibroblast activity. This therapy is particularly beneficial for managing chronic radiation injuries and for improving the outcomes of subsequent surgical interventions [231,232]. Photobiomodulation can be used in cancer patients for amelioration of radiodermatitis and fibrosis, mucosal necrosis, xerostomia, dysphagia, lymphedema, osteonecrosis, delayed fibrotic changes, and peripheral neuropathy [233].

### 3.3. Therapeutic Strategy—Surgical Approach

Surgical treatment addresses the improvement in irradiated tissue quality through methods such as regenerative therapy and lipofilling, as well as the need for surgical reconstruction in more severe cases [234].

Innovative therapies, such as cell injections, including autologous fibroblasts and multipotent stem cells, are being explored to enhance wound healing by promoting cellular interactions and the synthesis of growth factors. Research has continually explored the use of adult stem cells in regenerative medicine, including in wound healing after RT. Multipotent stem cell injections accelerate healing by synthesizing key growth factors, such as VEGF, PDGF, and TGF-β, and also promote angiogenesis and tissue regeneration [235,236]. Stem cell therapy and tissue engineering have the potential to enhance healing in irradiated tissues. Mesenchymal stem cells (MSCs) have shown promise in preclinical studies for their ability to promote angiogenesis, reduce inflammation, and enhance tissue repair. Research in this field is ongoing, with the aim of developing viable clinical applications, including the use of bioengineered tissue constructs and growth factors [237,238].

Autologous fat grafting and adipose-derived stem cell therapy are other techniques that are employed to restore volume and improve the quality of irradiated tissues. Fat grafting can enhance skin texture and elasticity, whereas stem cells may promote regeneration and repair of damaged tissues [239].

Adipose-derived stem cell therapy combined with angiogenic therapy has also been described. This innovative approach involves the use of adipose-derived stem cells combined with angiogenic deferoxamine to treat radiation-induced bone nonunion. Combination therapy has demonstrated significant improvements in biomechanical strength and healing, suggesting its potential for managing radiation-induced bony pathologies [240].

Surgical treatment is required in cases of severe and irreversible damage in which radiation injuries occur where tissue necrosis or loss of function occurs. This includes procedures such as debridement, skin grafting, use of negative pressure wound therapy, and the use of various flaps to restore function and cover defects resulting from the excision of necrotic areas. Key principles include thorough debridement, ensuring adequate blood supply to the affected area, and considering alternative solutions if the initial surgical interventions fail. These guidelines help in managing complex cases, from non-healing ulcers to radionecrosis and ORN [241].

Another crucial aspect for plastic surgeons is the influence of radiotherapy on complex reconstructive procedures following tumor excision, such as in areas like the head and neck or breast reconstructive surgery. The timing of reconstruction relative to RT is controversial, and there are many debates regarding the optimal time for reconstructive interventions. Immediate reconstruction can be complicated by acute radiation effects, whereas delayed reconstruction allows for the resolution of acute injuries but must contend with chronic changes. Optimal timing requires a balance between these factors, which are often individualized based on patient-specific considerations. Immediate reconstruction may be preferred because of its psychological benefits and reduced overall treatment time; however, delayed reconstruction allows for better assessment of radiation damage and patient recovery [242,243,244,245,246].

### 3.4. General Guidelines of Surgical Treatment for Radiation Ulcers

With adequate local wound management, most skin ulcers improve and, in some cases, may even heal by secondary intention. However, radiation ulcers progress and worsen because of underlying ischemia, infection, and lower viability of the granulation tissue [201,247].

Radiation ulcers can be treated with stable wound resurfacing after radical removal of nonviable tissue, such as skin, fat, muscle, and occasionally bone [201].

For more superficial lesions, special dressings such as hydrogel membranes, silver membranes, and skin allografts offer various benefits in wound healing. Hydrogel membranes help to maintain a moist environment, enhance re-epithelialization, and accumulate cytokines and growth factors that support healing. Silver dressings accelerate healing through their antimicrobial properties and improve the collagen organization. Skin allografts provide temporary dermal coverage, promote re-epithelialization, and protect the wound bed until definitive coverage can be achieved [236,248].

At the very least, flaps are required to treat radiation ulcers since the skin graft failure rate is nearly 100% because a previously irradiated wound bed does not have sufficient oxygen and nutrient supply [201,249].

This lack of oxygen and nutrient supply can be overcome or at least diminished by negative-pressure wound therapy (NPWT), which utilizes a vacuum dressing to manage complex wounds and enhance postoperative tissue repair. NPWT can be used only when malignancy is excluded. NPWT effectively removes wound exudate, reduces edema, and decreases bacterial load by applying adjustable negative pressure through an adhesive film and foam padding, leading to improved tissue perfusion and faster granulation. This therapy not only accelerates healing in chronic wounds but also helps prevent complications such as infections and wound dehiscence. Additionally, NPWT significantly benefits skin grafts by improving adherence and vascular integration, thereby promoting improved overall healing [250,251,252,253].

The use of tissue expanders in adjacent non-irradiated skin has been described by some researchers as a feasible novel method for treating radiation ulcers on the chest. This method has the advantage of not requiring extensive surgery and leaving the flap harvesting functionally intact. Additionally, because of the delay phenomenon, it is anticipated that the expanded skin will supply irradiated wounds with well-vascularized tissue [201].

Elevating the local flaps is also not recommended because radiotherapy often compromises the tissue surrounding the ulcer crater, resulting in flap loss. Reconstructive surgeons often prefer well-vascularized flaps to overcome the compromised blood supply in irradiated tissues. Because they provide large, well-vascularized tissues that support effective wound healing, axial musculocutaneous flaps, regional flaps (including perforator and propeller flaps), and microvascular free flap transfers are suggested for the resurfacing of radiation ulcers. The selection of the flap depends on several factors, including the size and location of the defect, availability of donor sites, and the patient’s overall health [201,249,254].

Alternatively, distal pedicled flaps harvested from nonirradiated areas produce satisfactory results. However, these patients may require staged surgery and extended hospitalization. The development of axial pattern flaps has historically marked significant advances in the reconstruction of post-radiation defects. The use of axial-pattern myocutaneous and muscle flaps allows for the treatment of these intricate ulcers. Large musculocutaneous flaps enable surgeons to perform a radical debridement. Furthermore, they are supplied by large blood vessels, which can promote healing in irradiated tissues [255,256].

**Figure 2:**
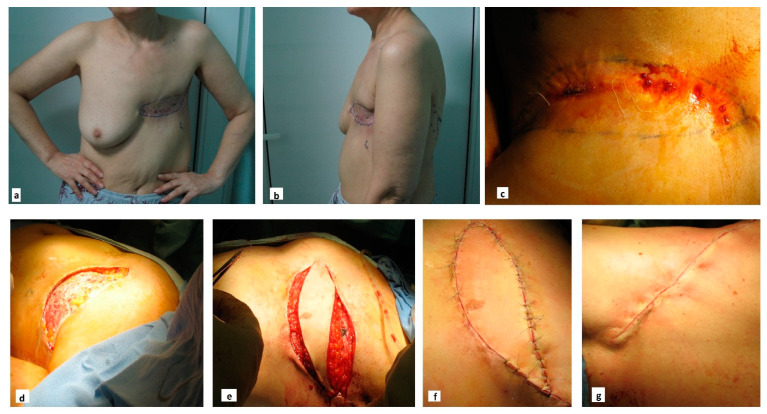
Female patient who underwent mastectomy for breast cancer, followed by radiotherapy, leading to a chronic ulceration of the postoperative scar and the surrounding tissues. (**a**–**c**): Preoperative aspect of the ulcerated area and planned resection markings, 6 months after completing radiotherapy. (**d**): Intraoperative aspect of the defect after resection of the ulcerated area. (**e**): Reconstruction of the defect with a pedicled latissimus dorsi myocutaneous flap. (**f**): Immediate postoperative aspect of the mammary region. (**g**): Immediate postoperative aspect of the donor site.

For example, the latissimus dorsi flap is ideal for large defects, providing reliable blood supply and substantial tissue volume. It can be used either as a pedicled flap or for free tissue transfer. The latissimus dorsi flap is a robust option for reconstructing defects in irradiated areas, especially in the chest wall and breast. It provides a well-vascularized tissue that can withstand the compromised vascular environment of the irradiated tissues. It is also used in combination with implants for breast reconstruction, offering a balance between aesthetic outcome and functional recovery [244,249,257]. Figure 2 illustrates the surgical treatment of a chronic radiation-induced lesion in a patient previously treated for breast cancer using a latissimus dorsi musculocutaneous flap.

The pedicled pectoralis major myocutaneous flap has been used as a reference for decades in head and neck reconstruction. The pectoralis major flap is no longer the first choice, particularly in developed countries where it is often replaced by a wide variety of free flaps that can address almost any defect in the head and neck region. Consequently, reconstruction is no longer a major concern in head and neck ablative surgeries. However, the pectoralis major flap is still frequently used, and although its indications have narrowed, it remains a valuable option in certain clinical settings [258].

The use of omentum flaps in the management of radiation-induced injuries has proven effective in various clinical scenarios. The rich vascular supply and immunological properties of the omentum make it suitable for reconstructing complex wounds and addressing complications from RT. Applications include chest wall reconstruction, spinal and sacral tumor surgery, and repair of pulmonary cutaneous fistulas. The benefits of omental flaps include enhanced vascularization, versatility in shaping, and reduced complication rates [259,260].

Advances in the understanding of perforator and angiosome anatomy have expanded the use of regional cutaneous and fascial flaps as effective alternatives to free flaps for skin defect reconstruction. Perforator flaps, which are supplied by isolated perforating vessels originating from the main arterial source, offer a robust option for the coverage of complex wounds. These flaps facilitate the use of regional tissues to achieve defect coverage, providing a well-vascularized skin paddle with the appropriate thickness and dimensions tailored to the wound. This method supports a single-stage reconstructive approach and enhances the potential for optimal aesthetic outcomes [261,262,263]. Consequently, when the radiation ulcer is relatively small and the perforator vessel and flap are outside the radiation field, perforator flaps have been used for radiation ulcer reconstruction [201]. Propeller flaps are increasingly used for small to moderate-sized defects, particularly in extremities and superficial sarcoma defects. They offer advantages such as less morbidity, faster recovery, and better aesthetic results than traditional free flaps [249,264].

The use of free microvascular transfers is an established treatment for radiation injuries, allowing surgeons to select the tissue that best fits the size and shape of the defect [232,265,266,267].

Imaging modalities, such as MRI and angio-CT, can provide detailed assessments of tissue viability and guide surgical planning, helping to optimize reconstructive strategies. Functional imaging techniques, such as dynamic contrast-enhanced magnetic resonance imaging (MRI), can assess tissue perfusion and identify areas of ischemia, guiding flap selection, and surgical planning [268,269,270].

However, it is not always simple to locate a suitable recipient vessel in the radiated region. Radiation-induced chronic endothelioangiitis in recipient vessels could be one of the causes of thrombosis [201]. A long vein graft was necessary for the free flap to reach and cover the wound [249]. As already mentioned, the latissimus dorsi flap can be used for free tissue transfer as well as for providing good blood supply and a large volume of tissue. The anterolateral thigh flap is versatile because of the possibility of considerable tissue harvesting with minimal donor-site morbidity. It is particularly useful in cases where there is significant tissue loss, such as radiation-induced ulcers with exposed vital structures. The radial forearm flap, as opposed to the latissimus dorsi and anterolateral thigh flaps, is suitable for smaller defects that require thin, pliable tissue [271].

The deep inferior epigastric artery flap is favored because of its muscle-sparing properties, making it an excellent choice for breast reconstruction after radiation. It provides a large volume of tissue with minimal donor-site morbidity [257,271].

Careful postoperative monitoring and management are essential for early detection and treatment of complications. The use of advanced wound care modalities and close collaboration with multidisciplinary teams including oncologists, radiologists, radiation physics specialists, and wound care specialists can improve outcomes. Regular follow-up is crucial to monitor the signs of wound dehiscence, infection, and other complications [201,236].

## 4. Conclusions

Radiotherapy, a crucial tool in the treatment of various malignancies, often leads to significant tissue damage and poses complex challenges for reconstructive surgery. The adverse effects that may occur after radiotherapy can significantly impact the patients’ quality of life and their recovery potential. This article highlights the multifaceted nature of radiation injuries and the diverse therapeutic approaches required to manage them, ranging from conservative measures to advanced surgical interventions, such as free tissue transfers. The intricate pathophysiological changes induced by radiation necessitate a tailored approach for each patient, with the primary goals being the restoration of function and aesthetics and the prevention of further complications.

The advancement of both prophylactic and therapeutic strategies, along with continuous innovation in reconstructive techniques, will be key to improving outcomes in patients affected by radiation-induced injuries. Interdisciplinary collaboration between oncologists, radiologists, and reconstructive surgeons is essential to optimize treatment plans and ensure comprehensive patient care.

## Figures and Tables

**Table 1:** Association between primary cancers and secondary radiation-induced malignancies [11,189,190].

Primary Cancer	Possible Associated Secondary Malignancies
**Lymphomas** **Hodkin Lymphoma** **Non-Hodkin Lymphoma**	Breast, thyroid, pulmonary, gastric cancer, colorectal, sarcoma Solid malignancies and leukemia
**Breast**	Lung, esophagus, leukemia, opposite breast cancer, sarcoma
**Testis**	Leukemia, lymphomas, pelvic malignancies, sarcoma of bone or soft tissue
**Prostate**	Bladder, rectal, lung, sarcoma
**Gynecological malignancies** **Cervical cancer** **Endometrial cancer**	Bladder, colo-rectal cancers, ovaries, uterus, leukemia, sarcomas Gastrointestinal malignancy-most encountered
**Pediatric cancers**	Thyroid, breast, leukemia, sarcoma

**Table 2:** Classification of radioprotector agents and their relevant representatives [205].

Radioprotectors Classification	Examples of Radioprotector Agents
Thiol-containing molecules	WR-2721 (amifostine) Dimethyl sulfoxide (DMSO) Glutathione (GSH) N-acetylcysteine (NAC) Erdosteine
Cyclic nitroxides	JP4-039 (gramicidin S-derived nitroxide)
Antimicrobials	Tetracycline, minocycline Ciprofloxacin Furazolidone
Phytochemicals	Plant extracts Polyphenolic phytochemicals Non-polyphenolic phytochemicals
Vitamins	Lutein Vitamin C, D2, D3, E
Oligoelements	Se, Zn, Mn
Superoxide dismutase mimetics and nanoparticles	EUK-207 Avasopasem manganese (GC4419) BMX-001
Hormonal and horomonal mimetics	Catecholamine agonists Somatostatin analogs Melatonin
Growth factors	Palifermin
Metformin	

## Data Availability

This paper is a literature review, no new data were created.
